# Genome-Wide Identification, Characterization, and Expression Analysis of the HD-Zip Gene Family in *Lagerstroemia* for Regulating Plant Height

**DOI:** 10.3390/genes15040428

**Published:** 2024-03-28

**Authors:** Hang Lin, Xinqiang Jiang, Cheng Qian, Yue Zhang, Xin Meng, Nairui Liu, Lulu Li, Jingcai Wang, Yiqian Ju

**Affiliations:** 1 College of Landscape Architecture and Forestry, Qingdao Agricultural University, Qingdao 266109, China; linhang202403@163.com (H.L.); jiangxinqiang8@163.com (X.J.); 20222110027@stu.qau.edu.cn (C.Q.); zy569980975@163.com (Y.Z.); m18895690475@163.com (X.M.); lnr15628611397@163.com (N.L.); yuanlinlilulu@126.com (L.L.); 2 East China Academy of Inventory and Planning of NFGA, Hangzhou 310019, China

**Keywords:** gene expression, HD-Zip gene family, *Lagerstroemia indica*, plant height

## Abstract

The Homeodomain leucine zipper (HD-Zip) family of transcription factors is crucial in helping plants adapt to environmental changes and promoting their growth and development. Despite research on the HD-Zip family in various plants, studies in *Lagerstroemia* (crape myrtle) have not been reported. This study aimed to address this gap by comprehensively analyzing the HD-Zip gene family in crape myrtle. This study identified 52 HD-Zip genes in the genome of *Lagerstroemia indica*, designated as *LinHDZ1-LinHDZ52*. These genes were distributed across 22 chromosomes and grouped into 4 clusters (HD-Zip I-IV) based on their phylogenetic relationships. Most gene structures and motifs within each cluster were conserved. Analysis of protein properties, gene structure, conserved motifs, and *cis*-acting regulatory elements revealed diverse roles of *LinHDZs* in various biological contexts. Examining the expression patterns of these 52 genes in 6 tissues (shoot apical meristem, tender shoot, and mature shoot) of non-dwarf and dwarf crape myrtles revealed that 2 *LinHDZs* (*LinHDZ24* and *LinHDZ14*) and 2 *LinHDZs* (*LinHDZ9* and *LinHDZ35*) were respectively upregulated in tender shoot of non-dwarf crape myrtles and tender and mature shoots of dwarf crape myrtles, which suggested the important roles of these genes in regulate the shoot development of *Lagerstroemia*. In addition, the expression levels of 2 *LinHDZs* (*LinHDZ23* and *LinHDZ34*) were significantly upregulated in the shoot apical meristem of non-dwarf crape myrtle. These genes were identified as key candidates for regulating *Lagerstroemia* plant height. This study enhanced the understanding of the functions of HD-Zip family members in the growth and development processes of woody plants and provided a theoretical basis for further studies on the molecular mechanisms underlying *Lagerstroemia* plant height.

## 1. Introduction

Transcription factors (TFs) play crucial roles in various aspects of plant growth [1], development [2], cell cycle and cell metabolism [3], and stress response [4]. One important TF family is the HD-Zip transcription factor family, which contains two highly conserved domains: the homeobox domain (HD) and the leucine zipper (LZ). This TF family is plant-specific [5]. The HD domain is a conserved motif comprising approximately 60 amino acid sequences, which fold to form a triple-helix DNA structure responsible for DNA binding [6]. The LZ domain mediates protein dimerization [3]. Based on the presence of conserved domains, functional characteristics, and motifs, the HD-Zip family can be classified into four subfamilies: HD-Zip I, II, III, and IV [7].

The protein structure of the HD-Zip I subfamily is relatively simple, comprising only the HD and LZ domains. Besides these domains, the HD-Zip II subfamily also possesses a conserved Cys, Pro, Ser, Cys, and Glu (CPSCE) motif, located adjacent to the LZ in the C-terminal direction [8]. Both the HD-Zip III and IV subfamilies contain START and SAD domains [9]. However, compared with the HD-Zip IV subfamily, proteins in the HD-Zip III subfamily have an additional methionine-glutamic acid-lysine-histidine-leucine-alanine (MEKHLA) domain at the C-terminus [10].

The HD-Zip gene family plays a crucial role in plant growth and development, with members of the same subfamily generally exhibiting similar gene functions and involvement in biological processes. HD-Zip I members are mainly associated with responses to abiotic stresses [11]. For instance, it has been reported that the expression of *LlHOX6* and *LlHB16*, members of the HD-Zip I subfamily, influences plant heat tolerance [12,13]. Additionally, Tang et al. revealed that the ectopic expression of *JcHDZ21*, a member of HD-Zip I, reduced the tolerance to salt stress in *Arabidopsis* [14]. Conversely, the expression of *MdHB7* enhanced the salt tolerance in apple plants [15]. HD-Zip II proteins mainly played essential roles in the auxin signal transduction pathway [16], organogenesis, and photosynthetic processes [17,18]. The overexpression of HD-Zip II gene *ATHB2* reduced auxin response and affected leaf development in *Arabidopsis* [19]. Sasake et al. reported that *EcHB1* belonging to the HD-Zip II subfamily increased photosynthesis and drought tolerance [20]. HD-Zip III was involved in various development processes, including shoot apical meristem development, vascular development, and regulation of plant stems and leaf polarity [21,22]. *PtrHB4*, a member of the HD-Zip III subfamily, affected the development of vascular cambium by regulating auxin signaling in poplar [23]. The HD-Zip III activator *ZIC2* promoted *Arabidopsis* shoot regeneration by limiting auxin transport [24]. HD-Zip IV genes were reported to play essential roles in regulating multiple physiological processes in plants, such as stomata, trichome, epidermis, and root hair development [19], as well as anthocyanin metabolism, trichome modeling, synthesis and transport of lipids, and protection of plants against biotic and abiotic stresses [25]. For instance, the HD-Zip IV gene *Roc8* was reported to regulate rice bulliform cell size and lignin content [26]. *OCL4* expression inhibited the development of maize epidermal hair and also affected cell division and differentiation to varying degrees [27].

Crape myrtle is a valuable ornamental plant with diverse plant types, abundant flowers, and a long blooming period [28]. The various plant types of crape myrtles cater to different landscaping needs. However, the molecular regulatory mechanisms governing plant types in *L. indica* remain unclear. Previous studies have identified internode length as a key factor affecting the *Lagerstroemia* plant height. Internode length is determined by cell division in the shoot apical meristem (SAM) and cell elongation in shoot segments. Studies have revealed that auxin and gibberellin are the primary hormones regulating internode length in crape myrtle [29,30,31]. Although the crucial roles of the HD-Zip family in auxin signal transduction and SAM development are increasingly recognized [12,32,33,34], their specific roles in *Lagerstroemia* have not yet been investigated.

This study involved an analysis of the HD-Zip gene family in *Lagerstroemia*, resulting in the identification of 52 HD-Zip genes within the genome of *L. indica*. Various aspects of these genes were investigated, including their chromosome location, protein physicochemical properties, gene structure, conserved domains, phylogenetic relationships, and the steady-state element of the HD-Zip gene family of *Lagerstroemia*. Additionally, the expression patterns of the 52 *LinHDZs* were examined across six tissues of non-dwarf and dwarf crape myrtles using transcriptome analysis (data not yet published). Further, a subset of 12 genes was selected for validation based on transcriptome data. These findings provide a theoretical basis for further exploration of the regulatory mechanisms of the HD-Zip family in controlling the plant height of crape myrtles.

## 2. Materials and Methods

### 2.1. Identification of HD-Zip Gene in L. indica

The genome database of *L. indica* was downloaded from CNCB (https://ngdc.cncb.ac.cn/gwh/Assembly/65978/show, access date: 5 December 2023) [35]. Representative domains of the HD-Zip gene family (PF00046 and PF02183) were acquired from the PFAM database (https://pfam.xfam.org/, access date: 5 December 2023) and used as queries. A preliminary search of the crape myrtle genome was conducted using the HMMER 3.0 tool, followed by manual removal of redundancy. Additionally, HD-Zip protein sequences of *Populus trichocarpa* were obtained from PlantTFDB (http://planttfdb.gao-lab.org/, access date: 5 December 2023). Those of *Oryza sativa* were obtained from NCBI (https://www.ncbi.nlm.nih.gov/, access date: 5 December 2023). Those of *Arabidopsis thaliana* were obtained from TAIR (https://www.arabidopsis.org/, access date: 5 December 2023). These protein sequences were further analyzed and validated using SMART (http://smart.embl-heidelberg.de/, access date: 7 December 2023) and CD-search (https://www.ncbi.nlm.nih.gov/cdd, access date: 7 December 2023) software to remove candidate sequences with mismatches or incomplete domains, resulting in the identification of putative *LinHDZs* in *L. indica*.

### 2.2. Analysis of Physicochemical Properties of HD-Zip Proteins in L. indica

The physical and chemical properties, such as relative molecular mass, theoretical isoelectric point, and number of amino acids of HD-Zip proteins, were predicted using TBtools software (version 2.052) [36]. The subcellular localization of the *L. indica* HD-Zip proteins was determined using the online tool Cello (http://cello.life.nctu.edu.tw/, access date: 7 December 2023).

### 2.3. Chromosome Localization Analysis of LinHDZ Genes

The chromosome distribution of the selected *LinHDZ* genes was predicted with Gene Location Visualize from GTF/GFF function of TBtools software.

### 2.4. Phylogenetic Tree Analysis of HD-Zip Genes in L. indica

*A. thaliana*, *O. sativa*, and *P. trichocarpa* were chosen as outgroup species, and the selected *LinHDZ* genes were used as the predicted population. Multiple sequence alignment analysis was performed using the ClustalW function of MEGA 6.0 software. The phylogenetic tree was constructed using the maximum likelihood method with the Jones–Taylor–Thornton (JTT) amino acid substitution model, and the bootstrap value was set to 1000 iterations [37]. The resulting phylogenetic tree was visually enhanced using iTOL (https://itol.embl.de, access date: 16 December 2023). The HD-Zip proteins used are presented in Table 1.

### 2.5. Prediction of Gene Structure, Conserved Motif, and Cis-Acting Regulatory Elements

The structures of the *LinHDZ* genes were predicted using GSDS (http://gsds.gao-lab.org, access date: 9 December 2023). Conserved motifs were analyzed using the MEME online tool with default parameters (https://meme-suite.org/meme/tools/fimo, access date: 9 December 2023) [38]. The 2000 bp upstream of the 5′ untranslated region of the *LinHDZ* genes was identified using the PlantCARE tool (http://bioinformatics.psb.ugent.be/webtools/plantcare/html, access date: 10 December 2023) to predict the *cis*-acting regulatory elements. The results were visualized using the visualization function of TBtools software.

### 2.6. Expression Profile Analysis of L. indica Using RNA-Seq Datasets

Transcriptome sequencing was conducted on six tissues—shoot apical meristem (SAM), tender shoot (TS), and mature shoot (MS) of both non-dwarf crape myrtle (S) and dwarf crape myrtle (D)—to investigate the regulation of HD-Zip genes on plant height in *Lagerstroemia*. A total of 3 μg of RNA for each sample was prepared for sequencing. Libraries were generated using an Illumina Novaseq platform. The gene expression levels were estimated using FPKM values. Differential expression analyses of S_TS vs. S_SAM, S_MS vs. S_SAM, D_TS vs. D_SAM, D_MS vs. D_SAM, and D_SAM vs. S_SAM were conducted using the DESeq2 R package (version 1.20.0), with genes having a *p*-value ≤ 0.05 and |log2 (fold change)| ≥ 1 considered as differentially expressed genes (Tables S2–S6). Three biological replicates were set for each tissue site. Tissue-specific expression patterns of *LinHDZ* genes were then analyzed.

### 2.7. Quantitative Real-Time–Polymerase Chain Reaction

The total RNA were extracted from different tissues of dwarf and non-dwarf crape myrtle using a total RNA extraction kit (Tiangen, Beijing, China) for qRT-PCR analysis, which was also used for the RNA-seq analysis. A total of 500 ng of RNA was reverse-transcribed into cDNA using the Prime Script RT reagent kit (TaKaRa, Dalian, China). The quantitative real-time polymerase chain reaction (qRT-PCR) was performed using the ACEX96 real-time PCR detection instrument (Bio-Rad, Hercules, CA, USA). *EF-1α* (Gen Bank ID: MG704141) was selected as the internal reference gene. The PCR system and procedures were conducted as previously described [39]. The expression levels were calculated using the 2^−ΔΔCt^ method, and the expression of each tissue was repeated three times biologically. All primer sequences are provided in Table S1.

## 3. Results

### 3.1. Genome-Wide Identification of HD-Zip Genes in L. indica

The genome of *L. indica* was analyzed using the BLAST tool in the PFAM database to identify potential HD-Zip genes. Then, the SMART and CD-search tools were used to confirm the existence of conserved HD and LZ domains, resulting in the identification of 52 HD-Zip genes designated as *LinHDZ1-52* (Table 1). The analysis revealed that the encoded proteins ranged in length from 222 (*LinHDZ16* and *LinHDZ52*) to 916 (*LinHDZ35*) amino acids, with an average length of 450 amino acids. The molecular weights of these *LinHDZs* ranged from 24.617 kDa (*LinHDZ52*) to 100.398 kDa (*LinHDZ35*), with an average value of 50.072 (Table 1). The isoelectric points of these proteins ranged from 4.63 (*LinHDZ33*) to 9.66 (*LinHDZ40*), with an average of 6.67. Subcellular localization analysis indicated that most *LinHDZs* were localized at the nucleus, whereas 10 were found in the cell membrane. Further details about *LinHDZs* are provided in Table 1.

### 3.2. Chromosome Localization Analysis of LinHDZ Genes

**Figure 1:**
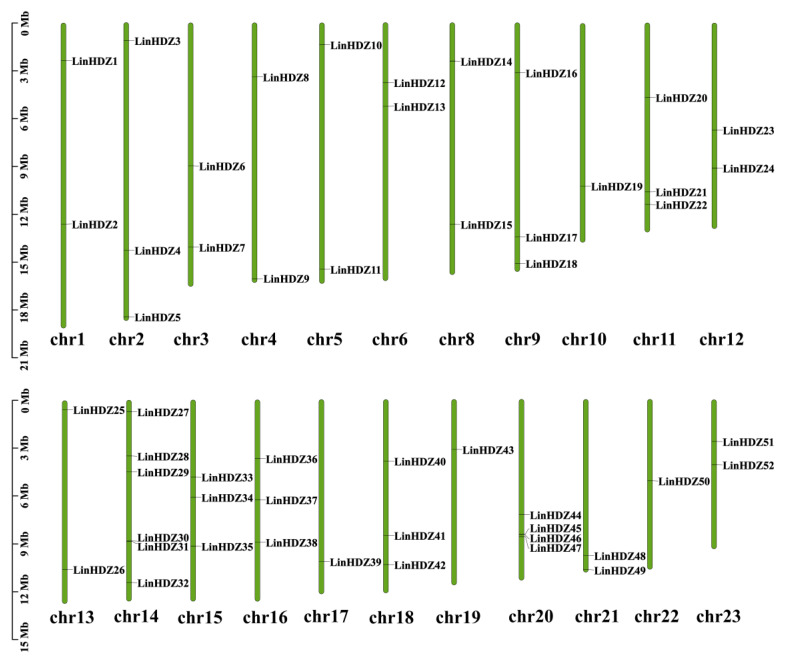
Localization of HD-Zip genes on the chromosomes of *Lagerstroemia indica*. Each chromosome is represented by a green strip, with the approximate distribution of each *LinHDZ* gene marked on the strip in black font.

The 52 *LinHDZs* were irregularly arranged across 22 chromosomes of the *L. indica* genome. Chromosome (Chr.) 14 harbored the highest number of HD-Zip genes, with six (11.54%), followed by four *LinHDZs* (7.69%) on Chr. 20. The results showed that Chr. 2, Chr. 9, Chr. 11, Chr. 15, Chr. 16, and Chr. 18 each contained three genes (5.77%); Chr. 1, Chr. 3, Chr. 4, Chr. 5, Chr. 6, Chr. 8, Chr. 12, Chr. 13, Chr. 21, and Chr. 23 each contained two genes (3.85%); and Chr. 10, Chr. 17, Chr. 19, and Chr. 22 each contained one gene (1.92%). No HD-Zip gene was localized on Chr. 7 and Chr. 24. (Figure 1). The uneven distribution of *LinHDZs* across chromosomes suggested the complexity and diversity of the HD-Zip family.

### 3.3. Phylogenetic Analysis of HD-Zip in L. indica

**Figure 2:**
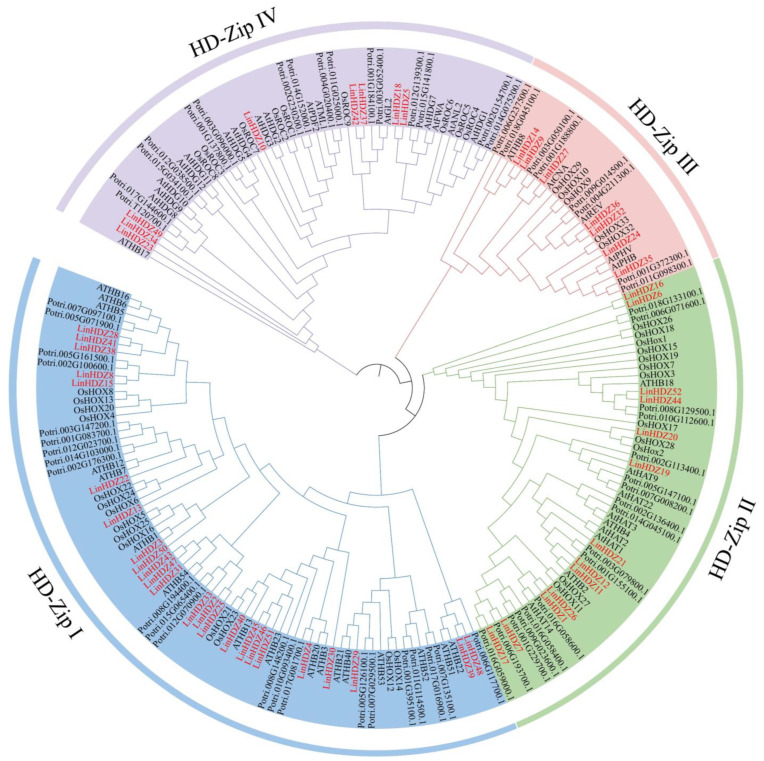
Phylogenetic tree analysis of HD-Zip sequences of *Lagerstroemia indica* and other plants. All *LinHDZ* genes in *Lagerstroemia indica* were marked in red font.

To elucidate the evolutionary relationship of HD-Zip genes between *L. indica* and other species, a phylogenetic tree was constructed using 48 *A. thaliana* HD-Zip, 40 *O. sativa* HD-Zip, and 63 *P. trichocarpa* HD-Zip proteins (Figure 2). The results revealed that the crape myrtle HD-Zip family could be categorized into four subfamilies (HD-Zip I–IV). The phylogenetic tree of HD-Zip genes from these four species demonstrated that the HD-Zip I subfamily had the largest number of representatives, followed by the HD-Zip II and IV subfamilies. Conversely, the HD-Zip III subfamily had the least number of representatives, with five in *A. thaliana*, five in *O. sativa*, eight in *P. trichocarpa*, and seven in *L. indica* (Figure 2). The phylogenetic tree with bootstrap values is shown in Figure S1. Generally, genes with close evolutionary relationships might have similar structures or biological functions.

### 3.4. Analysis of Conserved Motifs and Gene Structure of LinHDZs

**Figure 3:**
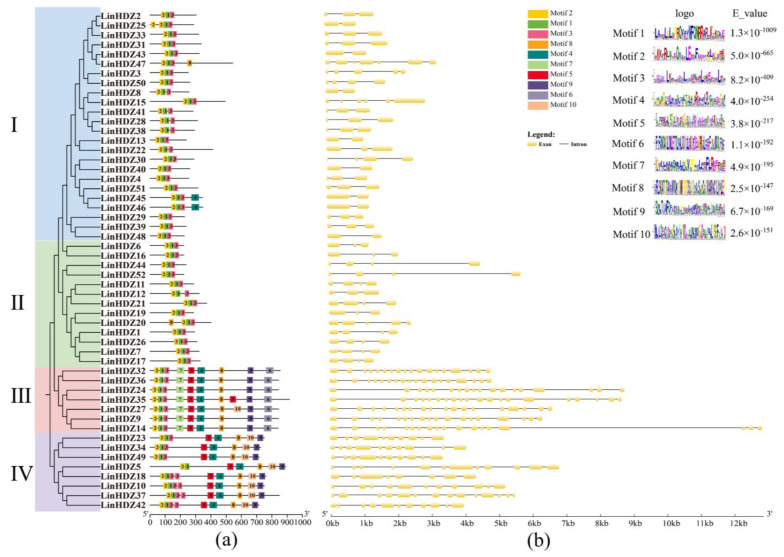
Gene structure and conserved motif analysis of *Lagerstroemia indica* HD-Zip family. (**a**) Distribution of conserved motifs in LinHDZ proteins; (**b**) Exon–intron structure of *LinHDZ* genes.

This study examined conserved motifs and gene structures to gain further insight into the evolutionary relationships and validate the classification accuracy of HD-Zip proteins in *L. indica*. Using the MEME online tool, the present study predicted the composition of conserved motifs in LinHDZ proteins, identifying 10 motifs. The motif composition within the same subfamily was largely consistent, indicating functional similarity among *LinHDZs* within the same subfamily due to the shared domain distribution. Conserved motif analysis revealed that all 52 *LinHDZs* contained motifs 1–3 corresponding to the HD and LZ domains, underscoring the importance of the two domains in *LinHDZ* expression. The high conservation of motifs 1–3 in *LinHDZs* aligned with the characteristic structural properties of HD-Zip proteins. Furthermore, HD-Zip I and II exclusively contained HD and LZ domains, whereas HD-Zip III and IV also included a START domain constituted by motifs 4, 5, and 8. In addition, the HD-Zip III subfamily featured a special MEKHLA domain, denoted by motif 6 (Figure 3a).

The analysis of gene structure revealed significant differences among the four subfamilies of HD-Zip genes, with members within the same subfamily exhibiting similar numbers of exons and introns. The exon–intron structure of HD-Zip I and II subfamilies appeared simpler compared with that of HD-Zip III and IV subfamilies. Specifically, HD-Zip I and II subfamilies predominantly had 3 to 4 exons, whereas HD-Zip III and IV subfamilies exceeded 10 exons (Figure 3b). These findings suggested that the HD-Zip gene family might have undergone exon supplementation or deletion during evolution.

### 3.5. Analysis of cis-Regulatory Element in LinHDZs

**Figure 4:**
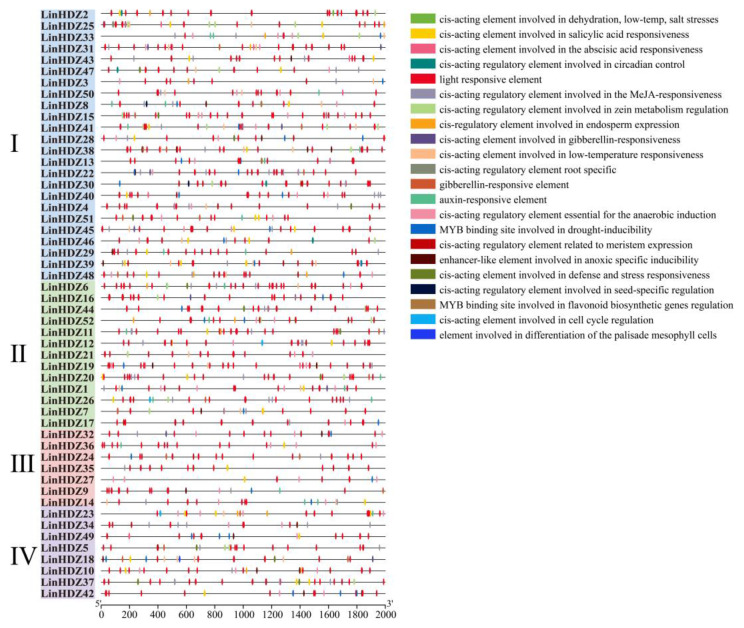
The distribution of 22 *cis*-acting elements in *LinHDZs* of *Lagerstroemia indica*. Segments of 200 bp are used as a ruler. Different color boxes represent different types of *cis*-acting elements.

As a regulatory factor controlling gene transcription and expression, *cis*-elements are indispensable in uncovering gene function [40]. The *cis*-regulatory elements of the *LinHDZs* (the 2 kb upstream of promoter region) were predicted to investigate the transcriptional characteristics and gene function using PlantCARE (Figure 4). Twenty-two *cis*-acting elements were detected in *LinHDZs*. Hormone-related elements, such as ABA, GA, IAA, SA, and MeJA response elements, were mainly distributed in HD-Zip I and II subfamilies. Among these, the *cis*-acting element involved in abscisic acid responsiveness was the most abundant (187), distributed among 52 *LinHDZ* genes. Stress response-related elements were mainly distributed in the HD-Zip I subfamily, with the *cis*-acting element involved in low-temperature responsiveness being the most prevalent (55), distributed among 29 *LinHDZ* genes. Functional elements related to growth and development were mainly distributed in HD-Zip II and III subfamilies, with the *cis*-acting regulatory element related to meristem expression having the largest number (33), distributed among 24 *LinHDZ* genes. The light-response element was present throughout the entire family. These results suggested that *LinHDZ* genes played crucial roles in affecting *Lagerstroemia* plant height and resisting external stress.

### 3.6. Analysis of Tissue-Specific Expression Patterns of LinHDZs in Non-Dwarf and Dwarf Crape Myrtles

**Figure 5:**
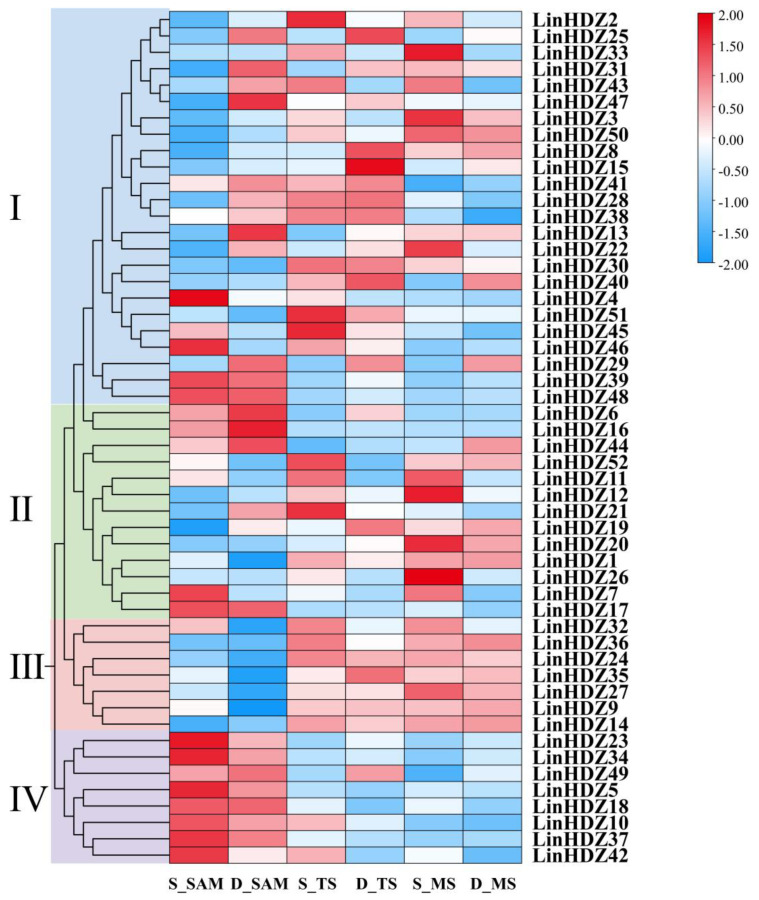
Cluster analysis of *LinHDZ* expression profiles in *Lagerstroemia indica*. The heat map was generated based on the log2-transformed relative expression values of *LinHDZs* in the shoot apical meristem (S_SAM), tender stem (S_TS), and mature stem (S_MS) of non-dwarf crape myrtle, and the shoot apical meristem (D_SAM), tender stem (D_TS), and mature stem (D_MS) of dwarf crape myrtle. Expression levels are depicted using a color gradient ranging from blue (downregulated) to red (upregulated).

The expression levels of 52 *LinHDZ*s in SAM, TS, and MS of non-dwarf and dwarf crape myrtles were analyzed using RNA-seq to investigate the mechanism of *LinHDZ* genes in regulating the plant height of *L. indica*. The expression patterns of *LinHDZs* across six tissues showed significant differences (Figure 5). The HD-Zip I subfamily exhibited expression in different tissues. In contrast, the HD-Zip II subfamily was mainly expressed in the shoot apical meristem of dwarf crape myrtle (D_SAM) and the TS and MS of non-dwarf crape myrtle (S_TS and S_MS). HD-Zip III and IV subfamilies showed tissue-specific expression patterns, with the HD-Zip III subfamily primarily expressed in TS and MS. The expression levels of *LinHDZ24*, and *LinHDZ14* in S_TS were significantly upregulated compared with those in S_SAM. The expression of *LinHDZ9* and *LinHDZ35* in both TS and MS of dwarf crape myrtles were about two times higher than those in D_SAM, while showed no significant differences in non-dwarf crape myrtles (Tables S2–S5). Additionally, the expression levels of *LinHDZ23* and *LinHDZ34* in the D_SAM were nearly two times lower than those in non-dwarf crape myrtle (Table S6). These results suggested that the effects of HD-Zip genes on the growth and development of *L. indica* varied among subfamilies, with HD-Zip III and IV subfamilies suggested to play essential roles in regulating *Lagerstroemia* plant height.

### 3.7. Validation of LinHDZ Expression

**Figure 6:**
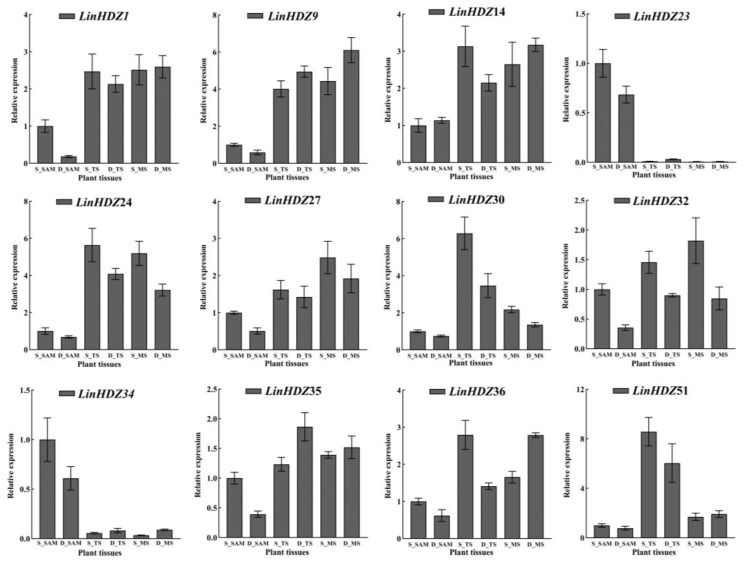
Expression pattern analysis of HD-Zip genes in the shoot apical meristem (S_SAM), tender stem (S_TS), and mature stem (S_MS) of non-dwarf crape myrtle, and the shoot apical meristem (D_SAM), tender stem (D_TS), and mature stem (D_MS) of dwarf crape myrtle. The error bar shows the standard error between three biological replicates (n=3). EF-1α (Gen Bank ID: MG704141) was selected as the internal reference gene and the expression levels were calculated by the 2^−ΔΔCt^ method to normalize qRT-PCR.

Twelve differentially expressed *LinHDZ* genes identified based on RNA-seq analysis (Figure 6) were subjected to qRT-PCR verification in SAM, TS, and MS tissues of both non-dwarf and dwarf crape myrtles. The results revealed significant upregulation of *LinHDZ24*, *LinHDZ14*, whereas *LinHDZ35* and *LinHDZ9* were markedly downregulated in S_TS. Additionally, *LinHDZ23* and *LinHDZ34* were significantly upregulated in S_SAM. The qRT-PCR results corroborated those obtained from RNA-seq analysis, indicating that these genes served as key candidates for regulating *Lagerstroemia* plant height. This study contributed to a deeper understanding of the molecular mechanisms underlying plant height regulation.

## 4. Discussion

Crape myrtle, being one of the most significant flowering plants in summer, has been widely used in gardens due to its diverse plant types. In recent years, dwarf crape myrtles have been increasingly favored by the garden market. However, the molecular mechanism behind the dwarf plant type of *Lagerstroemia* remains unclear. Identifying genes regulating plant height and exploring the regulatory mechanism of plant height can offer a crucial theoretical basis for enhancing plant types.

HD-Zip TFs play a crucial role in affecting plant growth, development, and resilience to environmental stress [41]. To date, HD-Zip family genes have been systematically identified in several species, such as Chinese cabbage, chrysanthemum, oil palm, and watermelon [42,43,44,45]. Various studies have demonstrated that HD-Zip III and IV genes mainly contributed to shoot and root meristem development, as well as cell proliferation regulation. However, the characteristics and functions of this gene family in crape myrtle have not yet been investigated.

This study identified 52 *LinHDZ* genes in the genome of *L. indica* and involved the genome-wide analyses of *LinHDZ*s. Phylogenetic tree analysis revealed that HD-Zip proteins were classified into four HD-Zip I–IV subfamilies, consistent with the findings in other species such as *A. thaliana* [46], *O. sativa* [47], and *P. trichocarpa* [48]. Most of these 52 *LinHDZ* genes belonged to HD-Zip I and II subfamilies. The HD-Zip III subfamily had the fewest *LinHDZ* genes (7/52), consistent with the proportions observed in other species such as *Prunus mume* (4/32) [49], *A. thaliana* (5/48) [46], and tomato (6/49) [50]. Analyses of gene-conserved motifs and gene structures revealed similar numbers of conserved motifs and exons and introns among members of the same subfamily, further supporting the reliability of the phylogenetic relationship of *LinHDZ* genes. Additionally, members of the same subfamily exhibited similar expression patterns across six tissues of non-dwarf and dwarf crape myrtles. HD-Zip I genes showed diverse expression across different tissues, whereas most HD-Zip II genes were expressed in S_TS and S_MS. HD-Zip III and IV subfamilies exhibited tissue-specific expression, with a preference for shoots and SAM, respectively. This might be because the members of HD-Zip III and IV subfamilies are known to be highly conserved lineages [45], with regulatory effects on SAM development, vascular development, leaf and shoot polarity regulation, and auxin transport [21,22,23,24,33]. These findings suggest that the four subfamilies of *LinHDZ* genes had different effects on the growth and development of *L. indica*. Previous studies have reported that HD-Zip gene members affect plant organ morphology. By combining tissue-specific expression patterns with previous findings, several members of HD-Zip III and IV subfamilies were implicated in regulating *Lagerstroemia* plant height. For instance, two *LinHDZ*s (*LinHDZ24*, and *LinHDZ14*) from the HD-Zip III subfamily were significantly upregulated in S_TS, with *LinHDZ14* being homologous to *ATHB8*, reported to be regulated by *AUX/IAA* involved in auxin signaling [51]. Studies on *Lagerstroemia* plant architecture highlighted the significance of IAA and GA hormones in regulating plant height [31]. Additionally, in *O. sativa*, the overexpression of *OsHox32*, a homologous gene of *LinHDZ35*, resulted in a semi-dwarf phenotype [52]. In this study, *LinHDZ35* was upregulated in D_TS and D_MS, suggesting its positive regulatory role in *Lagerstroemia* dwarfism. Previous studies have shown a complex regulatory relationship between the *ATML1* of HD-Zip IV and GA signal transduction, with negative feedback regulation between *ATML1*/*PDF2* and *DELLA* [53]. In the present study, *LinHDZ23* and *LinHDZ34* from the HD-Zip IV subfamily were homologous to *ATHDG11* and *ATHDG 12*, respectively. The latter were reported to play significant roles in regulating root and shoot meristems [54], suggesting their involvement in the cell proliferation of SAMs.

In summary, HD-Zip genes play a crucial role in the growth and development of crape myrtle. The findings of this study provide valuable insights into the role of HD-Zip genes in woody plants and might have significant implications for the breeding of *L. indica*, offering valuable references for future studies and applications in this area.

## 5. Conclusions

This study involved a comprehensive genome analysis of the HD-Zip family in *L. indica*. Fifty-two HD-Zip genes were identified and classified into four subfamilies: I, II, III, and IV. Gene structure and motif analysis revealed that the members within the same subfamily shared similar motifs and likely performed similar functions. *Cis*-acting element analysis indicated the presence of numerous hormone-related and stress-responsive *cis*-acting elements, suggesting their crucial role in regulating *Lagerstroemia* plant height and stress response. Tissue-specific expression profiling highlighted the significant impact of HD-Zip III and IV subfamilies on *Lagerstroemia* plant development. Moreover, six *LinHDZs* were identified as key candidates regulating the *Lagerstroemia* plant height, with *LinHDZ24* and *LinHDZ14* implicated in the positive regulation of branch elongation, *LinHDZ9* and *LinHDZ35* as negative regulators of shoot development, and *LinHDZ23* and *LinHDZ34* showing significant upregulation in S_SAM and playing roles in cell division. Overall, these findings not only enhance the understanding of the molecular mechanisms underlying HD-Zip family function in the growth and development of *Lagerstroemia*, but also provide insights for the molecular breeding of crape myrtle and other woody ornamental plants, as well as for further studies on these significant TFs.

## Figures and Tables

**Table 1:** Characteristics of HD-Zip gene family members in *Lagerstroemia indica*.

Sequence ID	Protein (aa)	MW (kDa)	pI	Instability Index	Aliphatic Index	GRAVY	Subcellular Localization
LinHDZ1	294	32.149	8.95	59.52	60.14	−0.94	Nuclear
LinHDZ2	304	34.653	5.31	59.16	64.14	−0.916	Nuclear
LinHDZ3	252	28.699	4.88	75.1	66.98	−0.812	Nuclear
LinHDZ4	256	28.853	8.41	57.74	71.33	−0.832	Nuclear
LinHDZ5	892	98.752	5.45	47.87	87.53	−0.188	PlasmaMembrane
LinHDZ6	223	25.027	8.77	43.7	80.04	−0.651	Nuclear
LinHDZ7	323	35.327	7.56	57.85	68.36	−0.637	Nuclear
LinHDZ8	257	29.454	5.13	62.97	69.46	−0.896	Nuclear
LinHDZ9	843	92.934	5.9	44.07	87.72	−0.114	PlasmaMembrane
LinHDZ10	752	82.448	5.4	36.15	80.52	−0.332	Nuclear
LinHDZ11	287	32.115	7.02	63.94	68.01	−0.731	Nuclear
LinHDZ12	325	36.207	8.21	66.93	74.18	−0.578	Nuclear
LinHDZ13	239	27.307	5.51	43.44	60.84	−0.956	Nuclear
LinHDZ14	841	92.753	6.06	44.43	86.41	−0.131	PlasmaMembrane
LinHDZ15	494	55.975	5.93	57.88	84.9	−0.397	Nuclear
LinHDZ16	222	24.711	6.61	52.84	64.68	−1.006	Nuclear
LinHDZ17	330	35.919	7.02	51.86	64.88	−0.748	Nuclear
LinHDZ18	762	84.141	5.6	51.79	81.13	−0.267	PlasmaMembrane
LinHDZ19	287	31.526	8.95	60.51	76.24	−0.543	Nuclear
LinHDZ20	401	45.821	9.41	56.35	67.66	−0.637	Nuclear
LinHDZ21	373	41.423	8.72	69.75	67.51	−0.708	Nuclear
LinHDZ22	413	46.682	6.29	50.16	79.64	−0.434	Nuclear
LinHDZ23	752	82.845	5.96	56.26	84.15	−0.297	Nuclear
LinHDZ24	845	92.835	5.87	52.08	84.84	−0.134	PlasmaMembrane
LinHDZ25	287	32.826	5.05	51.51	72.37	−0.723	Nuclear
LinHDZ26	309	33.907	5.64	57.92	62.27	−0.869	Nuclear
LinHDZ27	845	92.856	6.13	44.4	88.22	−0.096	PlasmaMembrane
LinHDZ28	313	34.628	5.02	57.55	69.23	−0.788	Nuclear
LinHDZ29	228	25.519	7.7	56.89	75.39	−0.684	Nuclear
LinHDZ30	290	32.999	6.17	61.97	59.59	−1.019	Nuclear
LinHDZ31	337	37.331	4.92	50.43	64.9	−0.753	Nuclear
LinHDZ32	855	93.606	5.92	50.04	88.9	−0.093	PlasmaMembrane
LinHDZ33	320	36.288	4.63	61.03	67.12	−0.785	Nuclear
LinHDZ34	727	80.399	5.78	50.14	80.62	−0.326	Nuclear
LinHDZ35	916	100.398	6.52	52.26	88.92	−0.127	PlasmaMembrane
LinHDZ36	844	91.863	5.61	47.3	87.51	−0.103	PlasmaMembrane
LinHDZ37	849	94.377	8.19	53.63	77.08	−0.449	Nuclear
LinHDZ38	293	32.639	4.92	58.05	72.29	−0.736	Nuclear
LinHDZ39	237	27.025	7.82	53.17	66.24	−0.748	Nuclear
LinHDZ40	262	29.574	9.66	61.81	78.17	−0.723	Nuclear
LinHDZ41	284	31.713	5.23	52.48	75.63	−0.676	Nuclear
LinHDZ42	717	80.437	7.16	55.17	75.61	−0.514	Nuclear
LinHDZ43	324	36.058	5.13	66.17	71.08	−0.716	Nuclear
LinHDZ44	238	26.377	8.88	85.03	63.99	−0.657	Nuclear
LinHDZ45	345	38.680	6.47	61.16	65.07	−0.632	Nuclear
LinHDZ46	347	38.920	6.57	59.42	66.11	−0.629	Nuclear
LinHDZ47	544	61.639	9.63	60.64	76.6	−0.606	Nuclear
LinHDZ48	226	25.663	9.12	61.26	75.58	−0.743	Nuclear
LinHDZ49	717	79.502	6.38	46.39	87.85	−0.245	PlasmaMembrane
LinHDZ50	262	30.172	4.96	67.21	57.67	−0.919	Nuclear
LinHDZ51	315	35.165	5.86	74.39	57.08	−0.845	Nuclear
LinHDZ52	222	24.617	8.73	88.6	65.09	−0.757	Nuclear

## Data Availability

Data are contained within the article and Supplementary Materials.

## Supplementary Materials

The following supporting information can be downloaded at: https://www.mdpi.com/article/10.3390/genes15040428/s1, Table S1. The primer sequences used in this study. Table S2. The differential expression levels of LinHDZ III subfamily in S_TS vs. S_SAM. The tender stem (S_TS) and shoot apical meristem (S_SAM) of non-dwarf crape myrtle. Table S3. The differential expression levels of LinHDZ III subfamily in S_MS vs. S_SAM. The mature stem (S_MS) of non-dwarf crape myrtle. Table S4. The differential expression levels of LinHDZ III subfamily in D_TS vs. D_SAM. The tender stem (D_TS) and shoot apical meristem (D_SAM) of dwarf crape myrtle. Table S5. The differential expression levels of the LinHDZ III subfamily in D_MS vs. D_SAM. The mature stem (D_MS) of dwarf crape myrtle. Table S6. The differential expression levels of LinHDZ III subfamily in D_SAM vs. S_SAM. The shoot apical meristem (S_SAM) of non-dwarf crape myrtle and the shoot apical meristem (D_SAM) of dwarf crape myrtle. Figure S1. Phylogenetic tree analysis of HD-Zip sequences of Lagerstroemia indica and other plants. All LinHDZ genes in Lagerstroemia indica are marked in red font. Numbers on the branch of the phylogenetic tree represent bootstrap values.
