# Supplementary material for: Genome-Wide Identification, Characterization, and Expression Analysis of the HD-Zip Gene Family in Lagerstroemia for Regulating Plant Height
Source: Genes (Basel). 2024 Mar 28;15(4):428. doi: 10.3390/genes15040428 (PMC11049174; doi:10.3390/genes15040428)
Supplement: Supplementary file 1 [file genes-15-00428-s001.zip › genes-2922560-supplementary.pdf]

Table S1. The primers sequences used in this study.

Name	Forward	Tm(℃)	GC%	Reverse	Tm(℃)	GC%
LinHDZ1	AGCCATACAACTCTTCAAGGG	55.90	47.6	AAAGAGATTGCTAGTCGGCG	58.23	50.0
LinHDZ9	AATGGACAACGGGAAGTACG	54.97	50.0	CGATGTTGGAGAGGATTGGG	56.10	55.0
LinHDZ14	AGAGATTGTACCACGAATGCC	55.82	56.7	ATGCCTCTTTCCTCTGCTTC	56.73	50.0
LinHDZ23	TGACCAGTTTGCATCTCAGAG	56.86	47.6	GTCCCTGTAGAACCGATGAAC	55.03	52.4
LinHDZ24	CGAGTCTGTGGTCTTGTTAGTC	56.74	50.0	CTCTATTGTCCCTCCATTTCCAG	57.17	47.8
LinHDZ27	CAGTTGGTGTACGAAAATGGC	55.21	47.6	CAGCTTGTGTCTTTGGTTGC	55.86	50.0
LinHDZ30	CTCCGACATCCTCAGACTAAATG	58.05	47.8	TGGTTGGGTTATGGTCGTG	53.29	50.0
LinHDZ32	CTTCACCATGTTCCCTGCTAG	57.01	52.4	ACCACAAGACTGCCATTATCC	54.94	47.6
LinHDZ34	GATACATCCTACATACCGCTGC	57.07	50.0	ACCACTGTCACCAACGAAC	53.35	50.0
LinHDZ35	GTGAGTCTGTGGTCATGAGTG	55.73	52.4	TTTGAGAGGAACTGTGCGAG	56.89	50.0
LinHDZ36	AGTGAATCGGAAACTGACGG	55.84	50.0	TGGTGTAGTGTGAAGTTGCTG	55.91	47.6
LinHDZ51	TCAACACCATCAGCATCCTC	55.07	50.0	GAATGACACCGATCTCTTCCC	55.69	52.4
EF-1α	GACTGTGCTGTGCTCATC	53.72	55.6	GTGGCATCCATCTTGTTG	50.89	50.0

Table S2. The differential expression levels of LinHDZ III subfamily in S_TS vs. S_SAM. The tender stem (S_TS) , shoot apical meristem (S_SAM) of non-dwarf crape myrtle.

subfamily	gene name	gene_id	S_TS1	S_TS2	S_TS3	S_SAM1	S_SAM2	S_SAM3	S_TS	S_SAM	log2FoldChange	pvalue
III	LinHDZ24	Lin_chr12_1084	1358.015	1376.526	1288.467	557.0692	516.4694	496.9492	1341.003	523.4959	1.356687	7.8×10^{-57}
III	LinHDZ14	Lin_chr8_0118	1168.682	1063.178	1058.236	418.7303	392.4811	380.0778	1096.699	397.0964	1.464749	2.8×10^{-54}
III	LinHDZ9	Lin_chr4_1747	796.7775	826.9164	864.1403	710.2632	710.9259	620.6954	829.2781	680.6282	0.284616	2.27×10^{-3}
III	LinHDZ35	Lin_chr15_0967	1950.809	1839.038	1970.901	1690.705	1771.517	1781.553	1920.249	1747.925	0.135466	3.74×10^{-2}

Table S3. The differential expression levels of LinHDZ III subfamily in S_MS vs. S_SAM. The mature stem (S_MS) of non-dwarf crape myrtle.

subfamily	gene name	gene_id	S_MS1	S_MS2	S_MS3	S_SAM1	S_SAM2	S_SAM3	S_MS	S_SAM	log2FoldChange	pvalue
III	LinHDZ14	Lin_chr8_0118	1160.541	1093.613	1113.499	430.8548	401.8452	388.5078	1122.551	407.0693	1.462839	1.54×10^{-58}
III	LinHDZ24	Lin_chr12_1084	1196.114	1154.596	1278.954	573.1993	528.7917	507.9714	1209.888	536.6542	1.1729	1.24×10^{-40}
III	LinHDZ35	Lin_chr15_0967	2182.129	2015.461	2131.908	1739.66	1813.783	1821.068	2109.832	1791.503	0.235764	9.71×10^{-05}
III	LinHDZ9	Lin_chr4_1747	1016.03	849.6848	803.5066	730.8291	727.8877	634.4623	889.7403	697.7264	0.348765	7.42×10^{-4}

Table S4. The differential expression levels of LinHDZ III subfamily in D_TS vs. D_SAM. The tender stem (D_TS), shoot apical meristem (D_SAM) of dwarf crape myrtle.

subfamily	gene name	gene_id	D_TS1	D_TS2	D_TS3	D_SAM1	D_SAM2	D_SAM3	D_TS	D_SAM	log2FoldChange	pvalue
III	LinHDZ9	Lin_chr4_1747	840.924	865.541	820.7082	176.424	169.2423	200.7617	842.3911	182.1427	2.210687	8.1×10^{-77}
III	LinHDZ24	Lin_chr12_1084	1078.878	1054.942	1146.768	313.0761	364.6562	347.6605	1093.529	341.7976	1.676443	3.03×10^{-60}
III	LinHDZ35	Lin_chr15_0967	2465.016	2446.935	2786.332	1074.861	855.8079	996.9533	2566.095	975.874	1.396642	5.82×10^{-54}
III	LinHDZ14	Lin_chr8_0118	833.9933	929.6928	997.6329	489.5002	539.1328	425.0273	920.4397	484.5534	0.925672	2.41×10^{-16}

Table S5. The differential expression levels of LinHDZ III subfamily in D_MS vs. D_SAM. The mature stem (D_MS) of dwarf crape myrtle.

subfamily	gene name	gene_id	D_MS1	D_MS2	D_MS3	D_SAM1	D_SAM2	D_SAM3	D_MS	D_SAM	log2FoldChange	pvalue
III	LinHDZ9	Lin_chr4_1747	1009.655	961.1549	973.6287	184.1189	176.6712	208.7511	981.4796	189.847	2.371106	2.25×10^{-95}
III	LinHDZ24	Lin_chr12_1084	1084.486	998.3888	1010.577	326.7311	380.6627	361.4957	1031.15	356.2965	1.530646	1.61×10^{-49}
III	LinHDZ35	Lin_chr15_0967	2214.764	2189.009	2189.916	1121.741	893.3736	1036.627	2197.896	1017.247	1.112874	1.84×10^{-37}
III	LinHDZ14	Lin_chr8_0118	1199.524	1126.543	1105.443	510.85	562.798	441.9413	1143.837	505.1964	1.177144	6.94×10^{-30}

Table S6. The differential expression levels of LinHDZ III subfamily in D_SAM vs. S_SAM. The shoot apical meristem (S_SAM) of non-dwarf crape myrtle and the shoot apical meristem (D_SAM) of dwarf crape myrtle.

subfamily	gene name	gene_id	D_SAM1	D_SAM2	D_SAM3	S_SAM1	S_SAM2	S_SAM3	D_SAM	S_SAM	log2FoldChange	pvalue
IV	LinHDZ23	Lin_chr12_0875	108.1562	87.31895	123.7056	405.5339	391.5854	437.6332	106.3936	411.5842	-1.95559	1.88×10^{-31}
IV	LinHDZ34	Lin_chr15_0706	224.0378	226.8394	190.8905	612.535	543.7186	551.02	213.9226	569.0912	-1.4107	2.63×10^{-26}
IV	LinHDZ42	Lin_chr18_1155	1354.16	1264.226	1133.613	2193.271	2056.949	2223.972	1250.666	2158.064	-0.78701	1.91×10^{-21}
IV	LinHDZ37	Lin_chr16_0678	578.3045	530.5575	524.6824	738.6175	772.3685	812.6053	544.5148	774.5304	-0.50871	6.55×10^{-07}
IV	LinHDZ10	Lin_chr5_0193	229.556	215.45	184.492	375.4247	314.1685	280.4831	209.8327	323.3588	-0.62441	1.29×10^{-4}
IV	LinHDZ5	Lin_chr2_1932	48.55992	21.82974	34.12568	57.39575	66.61453	41.77408	34.83845	55.26145	-0.67494	7.52×10^{-2}
IV	LinHDZ49	Lin_chr21_1054	75.04715	89.21718	125.8385	57.39575	103.5226	92.49974	96.70093	84.47269	0.194558	4.97×10^{-1}
IV	LinHDZ18	Lin_chr9_1614	50.76719	51.25243	63.98566	47.98661	69.31512	52.71491	55.33509	56.67221	-0.03673	9.07×10^{-1}

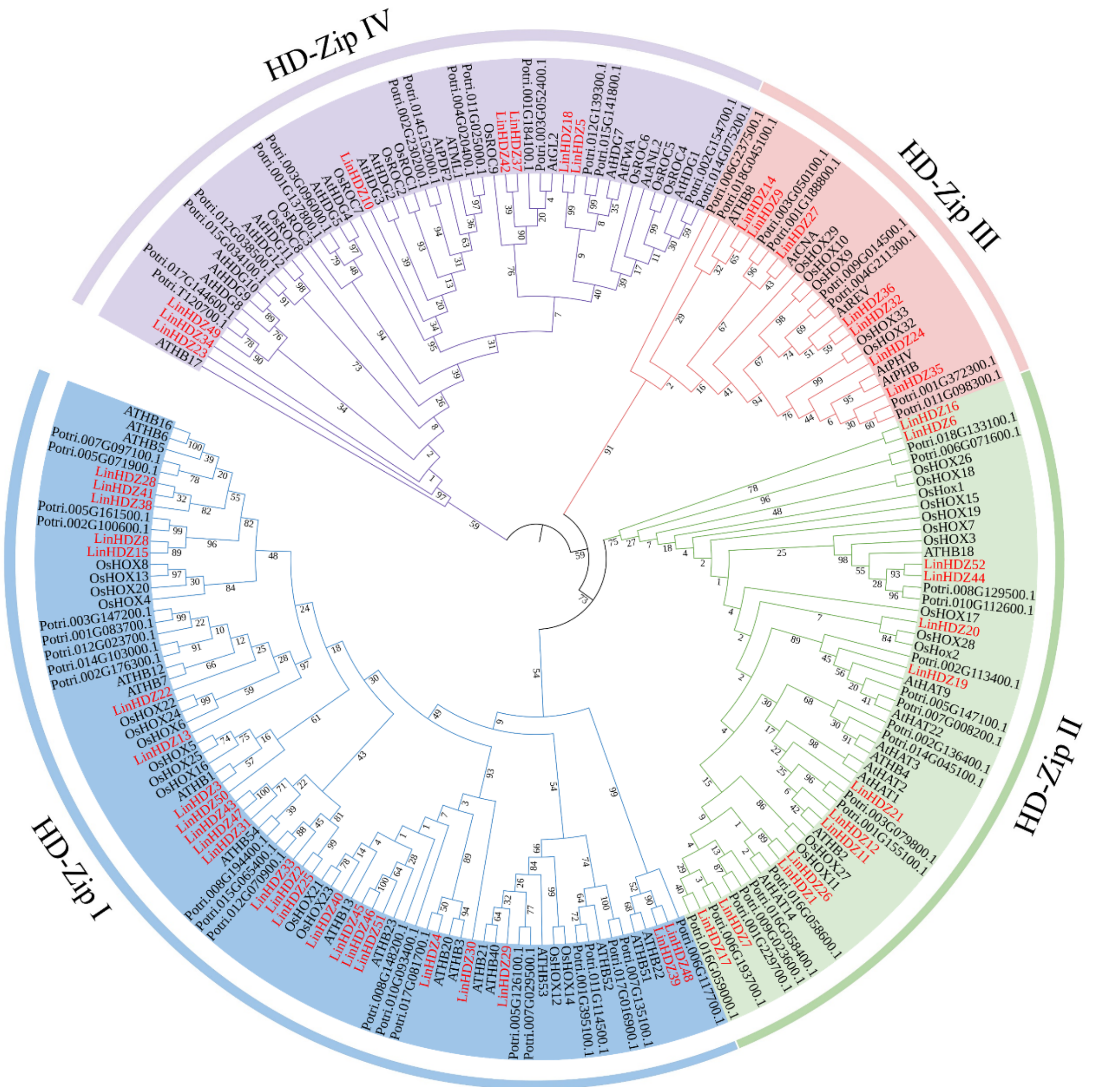

Figure S1. Phylogenetic tree analysis of HD-Zip sequences of *Lagerstroemia indica* and other plants. All *LinHDZ* genes in *Lagerstroemia indica* were marked in red font. Numbers on the branch of the phylogenetic tree represent bootstrap values.
